# Indoleamine 2,3-Dioxygenase Is Dispensable for The Immunomodulatory Function of Stem Cells from Human Exfoliated Deciduous Teeth

**DOI:** 10.22074/cellj.2016.4726

**Published:** 2016-09-26

**Authors:** Razieh Alipour, Masoumeh Masoumi Karimi, Batool Hashemi-Beni, Minoo Adib, Nasrin Sereshki, Farzaneh Sadeghi

**Affiliations:** 1 Department of Immunology, Medical School, Isfahan University of Medical Sciences, Isfahan, Iran; 2 School of Medicine, Shahroud University of Medical Science, Shahroud, Iran

**Keywords:** Immunomodulation, IDO, Mesenchymal Stem/Stromal Cells, SHED, IFN-γ

## Abstract

**Objective:**

In this study, we sought to better understand the immunoregulatory function of stem cells derived from human exfoliated deciduous teeth (SHED). We studied the role of the interferon gamma (IFN-γ)-indoleamine 2,3-dioxygenase (IDO)-axis in immunoregulation of SHED compared to bone marrow derived mesenchymal stem cells (BMMSCs) under the same conditions.

**Materials and Methods:**

In this cross-sectional study, recently isolated human T cells were stimulated either by mitogen or inactivated allogeneic peripheral blood mononuclear cells (PBMCs). These T cells were subsequently co-cultured with, either SHED or BMMSCs in the presence or absence of 1-methyl-tryptophan (1-MT) or neutralizing anti- human-IFN-γ antibodies. In all co-cultures we evaluated lymphocyte activation as well as IDO activity.

**Results:**

SHED, similar to conventional BMMSCs, had anti-proliferative effects on stimulated T cells and reduced their cytokine production. This property of SHED and BMMSCs was changed by IFN-γ neutralization. We detected IDO in the immunosuppressive supernatant of all co-cultures. Removal of IDO decreased the immunosuppression of BMMSCs.

**Conclusion:**

SHED, like BMMSCs, produced the IDO enzyme. Although IFN-γ is one of inducer of IDO production in SHED, these cells were not affected by IFN-γ in the same manner as BMMSCs. Unlike BMMSCs, the IDO enzyme did not contribute to their immunosuppression and might have other cell-type specific roles.

## Introduction

Mesenchymal stem/stromal cells (MSCs) are a rare stromal pluripotent, self-renewal cell population that belong to the adult stem cell subgroup. Initially, they have been introduced as non-hematopoietic bone marrow-derived cells (1,2). 

Diverse tissues and organs contain MSCs. The presence of MSCs in circulating blood, placenta, adipose tissues, dental pulp, and numerous other tissues have been shown (3,4). The differentiation abilities (5), angiogenesis, tissue remodeling/repair properties, growth factor release, and support of hematopoiesis are described as other characteristics of MSCs (3). The most intriguing features of MSCs considered superior to these attributes are their unique immune properties. 

MSCs have the capacity to modulate functions of various immune cells, as demonstrated both *in vitro* and *in vivo* in animal models and humans (2,6). MSCs can suppress human CD3+, CD4+, and CD8+ T lymphocytes (7, 8). They possess the ability to suppress proliferation of activated B cells and can alter the natural killer (NK) cell phenotype as well as suppress their proliferation, cytokine secretion, and cytotoxicity. Mature dendritic cells (DCs) are less functional in the presence of MSCs (9, 10).

Despite extensive investigations the exact underlying mechanism of the immunomodulatory role of MSCs is unclear (11). Nevertheless, it can be concluded from studies that the immunosuppressive ability of MSCs is not innate but induced by pro-inflammatory cytokines, in particular interferon gamma (IFN-γ) (12). A single factor alone does not appear to be responsible for immunosuppression; rather, multiple factors (although not equally) may be involved. Some of the suggested MSC-derived soluble factors involved in their immunosuppression include indoleamine 2,3-dioxygenase (IDO), transforming growth factor-β1 (TGF-β1), prostaglandin- E2 (PGE2), interleukin-10 (IL-10), and human leukocyte antigen G (HLA-G) (13-15).

Amongst these, there is more agreement on the role of the IFN-γ-IDO axis. Numerous studies have established that upon stimulation with IFN-γ, MSCs express IDO, an evolutionally conserved enzyme that degrades tryptophan through the kynurenine (KYN) pathway. IDO causes local deprivation of tryptophan and results in KYN metabolites that have immunosuppressive effects (16, 17).

Due to their remarkable capabilities, MSCs have tremendous potential in many modern therapeutic areas such as regenerative medicine, tissue engineering, and in novel immunosuppressant therapies. Thus, MSCs are the subject of numerous studies. In the majority of these studies, the classical bone marrow derived MSCs (BMMSCs) have been used. However, MSCs are relatively rare in human bone marrow. Their numbers and differentiation capacity decline with age. In addition, bone marrow aspiration is an invasive procedure (18). Regarding these limitations, conversely the promising results from clinical trials and consequently large numbers of MSCs required for future cellbased therapies, necessitates finding alternative sources for MSCs.

In recent years, stem cells from human exfoliated deciduous teeth (SHED) have emerged as a new population of MSCs (19) which can be a preferable source for their ever-increasing applications. exfoliated deciduous teeth are readily accessible and free from ethical concerns as the teeth are discarded. An advantage is the lack of pain and trauma to the patient when they are harvested. Obtaining stem cells from exfoliated deciduous teeth requires a simple, convenient method (20, 21).

Approximately 15 years ago Miura et al. (22) isolated SHED. Therefore, there is limited data; particularly the published studies on the immune properties of SHED are few (23). We have previously shown that SHED are comparable to conventional MSCs or BMMSCs. They suppress the proliferation and cytokine production of *in vitro* activated T cells (24). In the current study, we have examined the probable role of the IFN-γ-IDO axis in immunosuppression of SHED compared to BMMSCs.

## Materials and Methods

Approximately 10^5^ T cells stimulated by phytohemagglutinin (PHA) in the lymphocyte transformation test (LTT) or allogeneic peripheral blood mononuclear cells (PBMCs) in MLC were placed adjacent to various numbers of SHED or BMMSCs, as separate co-cultures. MSCs were inactivated by mitomycin in order to prevent expansion and consequently delete any further interference with lymphocyte proliferation. Previous studies demonstrated that the dose of MSCs might affect their immunoregulation, therefore we used a different number of MSCs.

The Ethics Committee of Isfahan University of Medical Sciences approved this analytical-descriptive, cross-sectional study.

### In vitro expansion of human stem cells

One T25 cell culture flask of passage 2 SHED was kindly provided by the Torabi Nejad Research Center, Dental School, Isfahan University of Medical Sciences, Isfahan, Iran for this research.

Passage 3 human BMMSCs were purchased as a 70% confluent T75 cell culture flask from Royan Institute, Isfahan, Iran. In order to obtain an adequate number of cells to culture with the lymphocytes, we expanded both BMMSCs and SHED in vitro as previously described (24).

### Isolation of human blood cells

T Lymphocytes were isolated from the buffy-coat of a healthy volunteer using the RosetteSep® Human T Cell Enrichment Cocktail (StemCell Technologies, Vancouver, BC, Canada). The lymphocytes were suspended in complete RPMI1640 that consisted of RPMI1640 with L-glutamine (Sigma, Germany) supplemented with 10% fetal calf serum (FCS, Gibco. Germany), and 1% penicillin/streptomycin (Roche, Germany).

PBMCs were obtained by Ficoll density gradient from heparinized blood samples of a healthy volunteer donor who was allogeneic to the person who donated the T lymphocytes. The isolated cells were suspended in complete RPMI1640.

### Simple co-cultures

#### Direct co-cultures

Both types of human MSCs were mitomycin C inactivated by incubation for 3 hours in 7.5 μg/ml. Then, 100 μl aliquots of completed RPMI1640 plus decreasing numbers (104, 4×10^3^, 2×10^3^ and 103 cells) of viable MSCs either SHED or BMMSCs were plated in flat-bottomed 96-well plates, then allowed to adhere for 12 to 16 hours. Completed RPMI1640 without MSCs was used as the control

Next, we performed the mitogen proliferation assay (lymphocyte transformation test, LTT) as follows: 10^5^ T cells in 100 μl completed RPMI1640 stimulated by 4 μg/ml PHA (Roche, Germany) were added to the MSCs. Controls consisted of 10^5^ PHA-stimulated T cells (positive control) or unstimulated 10^5^ T cells (negative control) added to wells that contained only complete medium. Additionally, as background controls, we added 100 μl medium (without T cells) to wells that contained only the co-cultures of diminishing numbers of MSCs plus SHED or BMMSCs. The cultures were incubated for 3 days at 37˚C in a humidified atmosphere of 5% CO_2_.

In the mixed lymphocyte cultures (MLCs), once the MSCs adhered to the plates we added responder cells (10^5^ T cells) in 50 μl of completed RPMI1640, followed by stimulator cells that consisted of an equal number of mitomycin inactivated PBMCs (25 μg/ml for 40 minutes) in another 50 μl of medium. The positive control consisted of responder plus stimulator cells, whereas the negative control consisted of only responder cells. Cultures were used as background controls in the same manner as the LTTs. The cultures were incubated for 5 days at 37˚C in a humidified atmosphere of 5% CO_2_.

### Indirect co-cultures

Transwell analysis was performed with a 24-well transwell insert system (Costar) with a pore size of 0.4 μm. The lower chambers contained 2.5×10^4^, 104, 5×10^3^, and 2.5×10^3^ inactivated MSCs with either SHED or BMMSCs. For the LTTs, 2.5×10^5^ PHA-stimulated human T cells, and for MLCs, 2.5×10^5^ T cells (plus an equal number of stimulator cells) were cultured in the upper chambers of the wells. Appropriate controls included positive, negative and background controls were set. All transwell cultures were performed in a total volume of 500 μl of completed RPMI 1640 medium.

All cultures were established in triplicate. After incubation, we assessed T lymphocyte proliferation and cytokine secretion.

### Extra co-cultures

In order to investigate the role of IFN-γ and IDO in suppression of MSCs, at the beginning of the culture periods we added either neutralizing monoclonal anti-human IFN-γ antibodies (10 mg/ml, R&D Systems, USA), as the Ab-co-cultures, or 1-methyl-tryptophan (1-MT, Sigma Aldrich, Germany) as the MT-co-cultures. The concentrations were selected from literature searches of previous studies followed by in-house optimization.

### Proliferation assays and cytokine analysis

T cell proliferation was evaluated by measuring BrdU (a thymidine analogue) incorporation into the DNA of proliferating T cells by a Cell Proliferation ELISA, BrdU (colorimetric) kit (Roche, Germany) according to the manufacturer’s protocol. The optical density (OD) of each co-culture/culture indicated lymphocyte proliferation.

To eliminate the effect of probable BrdU incorporation in MSCs co-cultured with stimulated lymphocytes, we used a set of cultures which contained only MSCs; the numbers of MSCs in these cultures were the same as the co-cultures (namely background controls). We subtracted the absorbance of these cultures from the absorbance of the corresponding co-cultures (numbers and MSC type) and this OD was used.

The amount of interleukin-2 (IL-2) and IFN-γ were assayed in pooled supernatant of three repeats of each culture with the Human IL-2 Enzyme- linked Immunosorbent Assay (ELISA) Kit and Human IFN-γ ELISA Kit (both from R&D Systems, USA).

### Kynurenine assay to assess indoleamine 2,3-dioxygenase activity

We mixed 100 μl of each culture supernatant with 50 μl of 30% trichloroacetic acid (Merck, Germany). The solution was vortexed and subsequently incubated at a temperature 56˚C for 20 minutes, then centrifuged at 800 g for 5 minutes. We transferred 75 μl of the supernatant to 96-well flat-bottom plates and added an equal volume of Ehrlich reagent (100 mg of p-dimethylbenzaldehyde (Sigma Aldrich, Germany) in 5 ml of glacial acetic acid (Merck, Germany). The absorbance was read at 450 nm in a microplate reader. The concentration of KYN was calculated using a standard curve of L-KYN (Sigma- Aldrich, Germany) concentration (0-100 μM).

### Statistical analysis

Statistical analysis was done by the Statistical Package for the Social Sciences (SPSS) software version 16. Statistical significance was calculated using t test analyses and univariate analysis of variance. Significance was set at P<0.05.

## Results

We examined the propagation of stimulated T lymphocytes as a marker of their activation. Initially, the proliferation of each culture/co-culture was calculated by dividing the obtained OD by the OD of unstimulated T cells.

Proliferation (P)=OD of the stimulated T cells with or without MSCs (cultures or co-cultures)/ OD of unstimulated T cells (negative controls) Then, to simplify the comparison of the inhibitory effect of MSCs on T cells, we calculated the proliferation inhibition (PI) by the following formula:

PI=1-[proliferation (P) of intended co-cultures/ proliferation (P) of the corresponding positive control (LTT or MLC without MSCs)]×100.

### Increased immunosuppression of human exfoliated deciduous teeth and bone marrow derived mesenchymal stem cells were accompanied by increased indoleamine 2,3-dioxygenase activity

Initially we examined the presence of functional IDO in the supernatants of the cultures/co-cultures. IDO activity was assessed by measuring the quantity of KYN.

**Fig.1:**
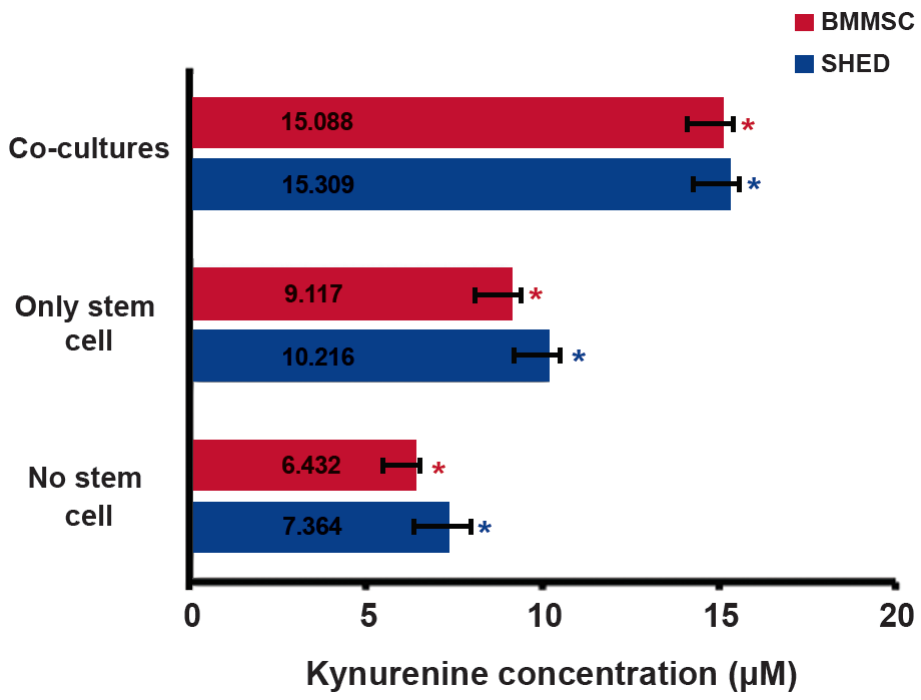
The results showed increased indoleamine 2,3-dioxygenase (IDO) activity in the presence of mesenchymal stem/stromal cells (MSCs). We assessed the kynurenine (KYN) concentration (IDO activity) in all cultures. The results are reported as means ± SEM of at least two experiments (each performed in triplicate). The co-cultures consisted of stimulated T cells and MSCs, whereas the "No stem cell" group comprised the positive and negative cultures that contained only lymphocytes. The “Only stem cell” group consisted of background controls with only MSCs. Asterisks indicate significant differences (one-way ANOVA, P<0.05) between groups. *; Bone marrow derived mesenchymal stem cells (BMMSCs) and *; Stem cells derived from human exfoliated deciduous teeth (SHED).

We found a basal KYN concentration even in the absence of MSCs (in positive and negative controls). Markedly elevated IDO activity existed in cultures that contained only stem cells (in background controls). We observed the highest enzyme activity in the immunosuppressant supernatants of the MSC co-cultures (Fig .1).

**Fig.2:**
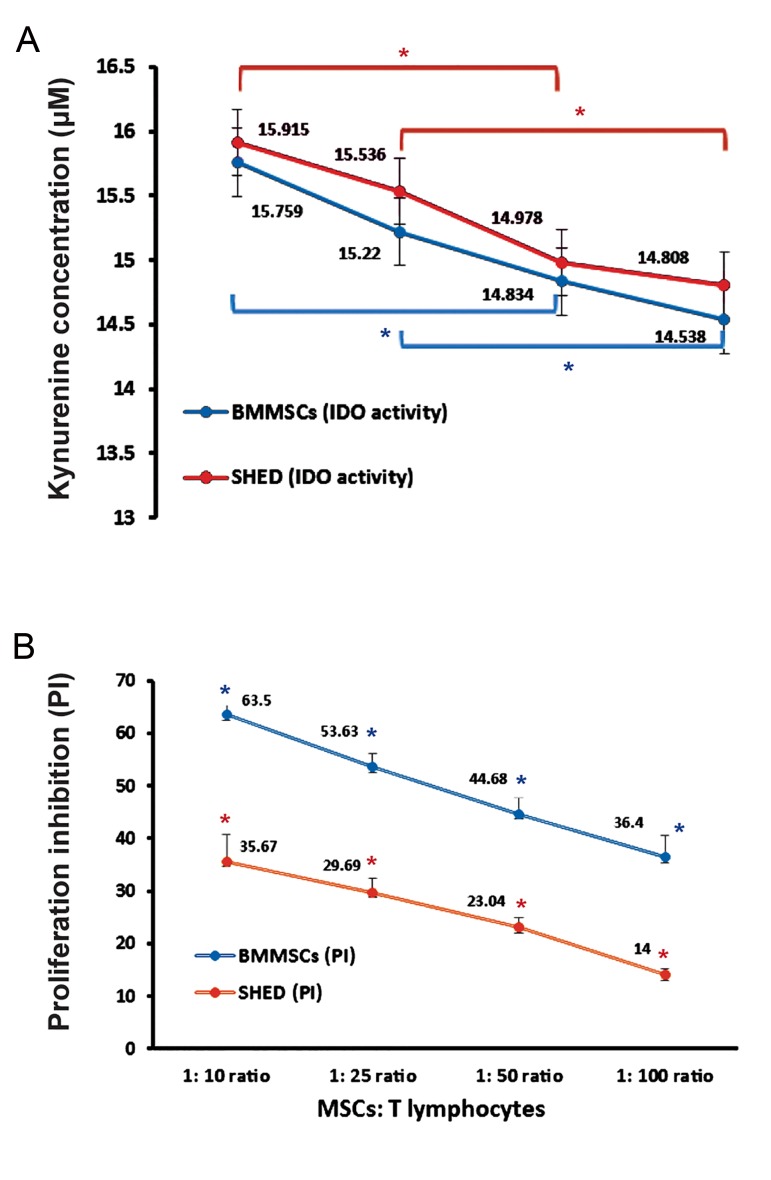
Elevation in indoleamine 2,3-dioxygenase (IDO) activity accompanied by inhibition of mesenchymal stem/stromal cells (MSCs). A. The kynurenine (KYN) concentrations were measured in the co-cultures that contained different numbers of MSCs. The horizontal axis showed the ratio of stimulated T cells to MSCs [stem cells derived from human exfoliated deciduous teeth (SHED) or bone marrow mesenchymal stem cells (BMMSCs)] in the co-cultures (identical to the horizontal axis of graph B) and B. Proliferation of stimulated T cells in the presence of different number of MSCs. Proliferation inhibition (PI) calculation was calculated as detailed in the text. Results are mean ± SEM of at least two experiments (each performed in triplicate). Asterisks indicate significant differences (one-way ANOVA, P<0.05) between groups. *; BMMSCs and *; SHED.

We assessed IDO activity in the immunosuppressive environment of the co-cultures. The results revealed that the amount of KYN (IDO activity) was proportional to the increase in suppression of MSCs as a result of their increased numbers. This supported the fact that IDO might be a key mediator in the immunoinhibitory effects of both BMMSCs and SHED. Figure 2A and B show the parallel relationship between IDO activity and PI.

### 1-methyl-tryptophan and neutralizing anti-interferon gamma antibody decreased bone marrow derived mesenchymal stem cell immunosuppression

**Fig.3:**
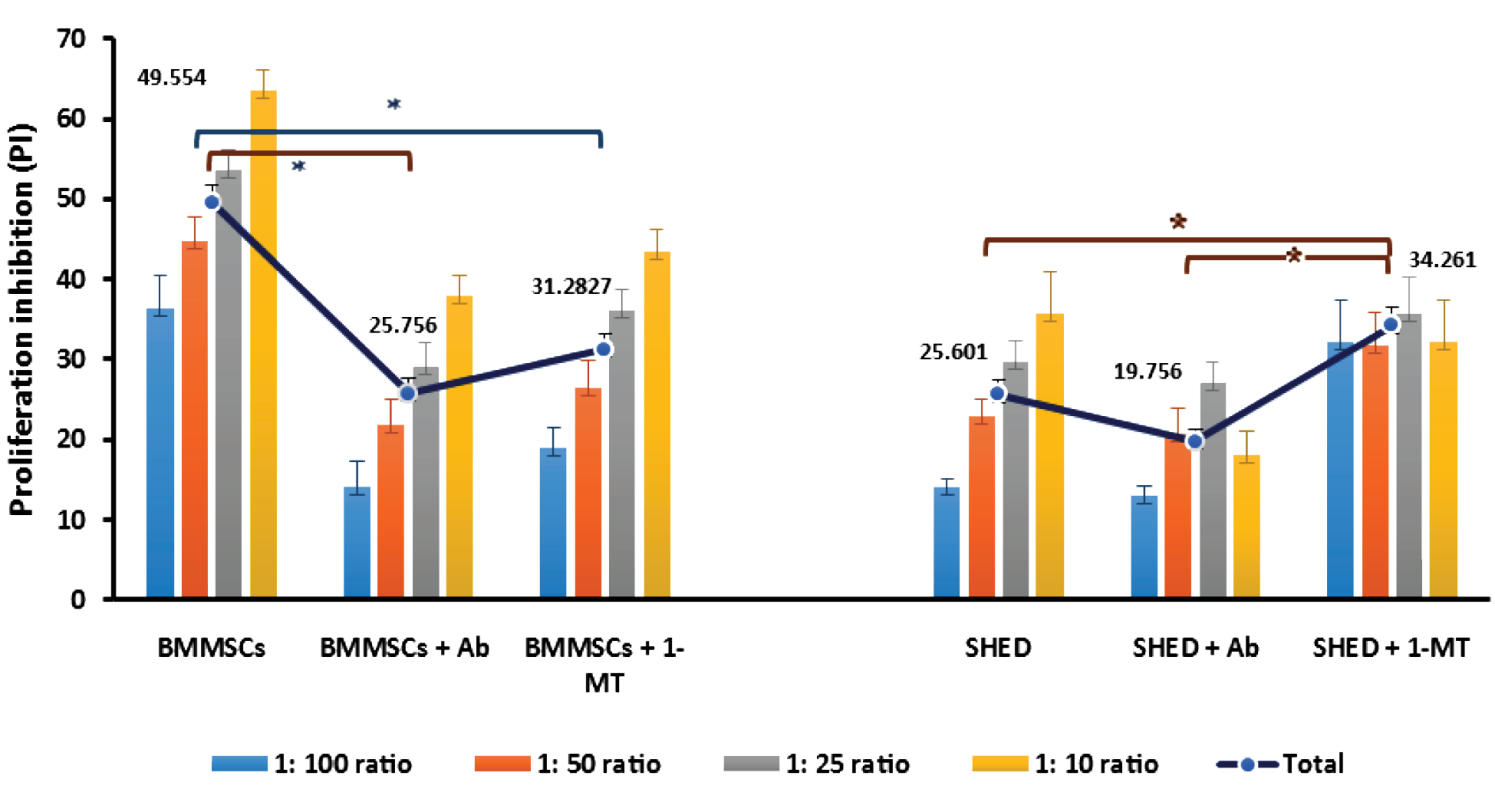
Inhibition of stem cells derived from human exfoliated deciduous teeth (SHED) and bone marrow mesenchymal stem cells (BMMSCs) on stimulated T lymphocytes in three set of co-cultures: 1 mM 1-methyl tryptophan (1-MT), 4 μg/ml neutralizing anti-interferon gamma (anti-IFN-γ) antibody, and simple co-cultures (without any exogenous factor). The results are presented as the means and SEM from at least two experiments (each performed in triplicate). Total inhibition for three sets of co-cultures are shown by the solid line whereas the columns show inhibition of co-cultures with different numbers of mesenchymal stem/stromal cells (MSCs). Asterisks indicate significant differences (one-way ANOVA, P<0.05) between groups. *; BMMSCs and *; SHED.

We used 1-MT, as an inhibitor of IDO, to further elucidate whether the suppressive effect of MSCs was attributed to IDO. We added 1 mM of 1-MT to the co-cultures, which were named MT-co-cultures. The addition of 1-MT restored the activation of T cells in the BMMSC co-cultures. As the PI was markedly diminished (Fig .3), the IL-2 and IFN-γ levels increased significantly (Table 1) compared to the simple co-cultures. The level of KYN, as a marker of IDO activity, showed a remarkable drop in the MT-co-cultures (Table 1). We examined the role of IFN-γ, in extra immunosuppressant co-cultures, by adding neutralizing anti-human IFN-γ antibody at thte first of the cultures/co-cultures at a concentration of 4 μg/ml (Ab-co-cultures).

Results of the BMMSC co-cultures showed a notable decrease in the suppression of lymphocyte proliferation (Fig .3). This reduction occurred in conjunction with a remarkable diminish in IDO activity. In addition to the reduction in PI, we observed an elevation in IL-2 to some extent in the Ab-co-cultures. This supported the fact that Ab could reverse the activation of stimulated T cells. There was a substantial drop in the IFN-γ level (Table 1), more likely because of the presence of neutralizing antibody.

In terms of the Ab- and MT-co-cultures, we observed that lymphocyte activation in the Ab-co-cultures reversed more at all numbers of BMMSCs. However, PI (P=0.115), IL-2 quality (P=0.380), and IDO activity (P=0.134) did not show statistically significant changes compared to the MT-co-cultures (Table 1).

### Effects of 1-methyl-tryptophan and neutralizing anti-interferon gamma (anti-IFN-γ) antibody on human exfoliated deciduous teeth (SHED) immunosuppression

We had co-cultures of SHED and T cells; the simple co-cultures, the Ab-co-cultures with neutralizing anti-IFN-γ antibody (4 mg/ml), and the MT-co-cultures with 1-MT (1 mM) in the same manner as the BMMSC co-cultures.

We observed in the Ab-co-cultures that the antibody could not efficiently block the immunosuppressive effect of SHED since the PI (Fig .3) and IL-2 production (Table 1) changed slightly compared to the simple co-cultures. However, we observed considerably reduced IFN-γ levels and IDO activity (Table 1).

Surprisingly, the results of SHED and T lymphocyte MT-co-cultures had tremendous growth in PI compared to the simple co-cultures (Fig .3). In conjunction with the PI results, we observed reduced cytokine levels which was meaningful for IL-2 but not for IFN-γ (Table 1). There was significantly decreased IDO activity in the MT-co-cultures compared to the simple co-cultures (Table 1).

A comparison between MT- and Ab-co-cultures showed remarkable differences in PI (P<0.05) and cytokine (for both cytokines, P<0.05) levels.

**Table 1:** Concentrations of cytokines and indoleamine 2,3-dioxygenase (IDO) activity in supernatants of the Ab-, simple- and MT-co-cultures as determined by ELISA (cytokines) or colorimetric method (IDO). The corresponding P values (one-way ANOVA) are shown in the right of its column (the upper and lower p-values correspond to the differences between the simple co-cultures and either the Ab- co-cultures or MT-co-cultures).


Co-cultures	BMMSCs						SHED					
	IL-2 (pg/ml)	P value	IFN-γ (pg/ml)	P value	IDO activity (μM)	P value	IL-2 (pg/ml)	P value	IFN-γ (pg/ml)	P value	IDO activity (μM)	P value

Ab	44.83±1.96	0.058	15.82±0.77	0.056	13.96±0.30	<0.05	67.95±4.08	0.593	28.69±1.08	<0.05	13.92±0.25	<0.05
Simple	37.02 ± 0.48		25.15±1.92		14.93±0.31		65.19±3.11		44.96±2.50		15.33±0.22
MT	48.44±1.96	<0.05	62.83±4.73	<0.05	13.15±0.27	<0.05	38.42±1.37	<0.05	38.15±1.77	<0.05	12.46±0.30	<0.05


BMMSCs; Bone marrow mesenchymal stem cells, SHED; Stem cells derived from human exfoliated deciduous teeth, and IFN-γ; Interferon gamma.

### Physical contact effectively suppressed mesenchymal stem cells under different conditions

**Fig.4:**
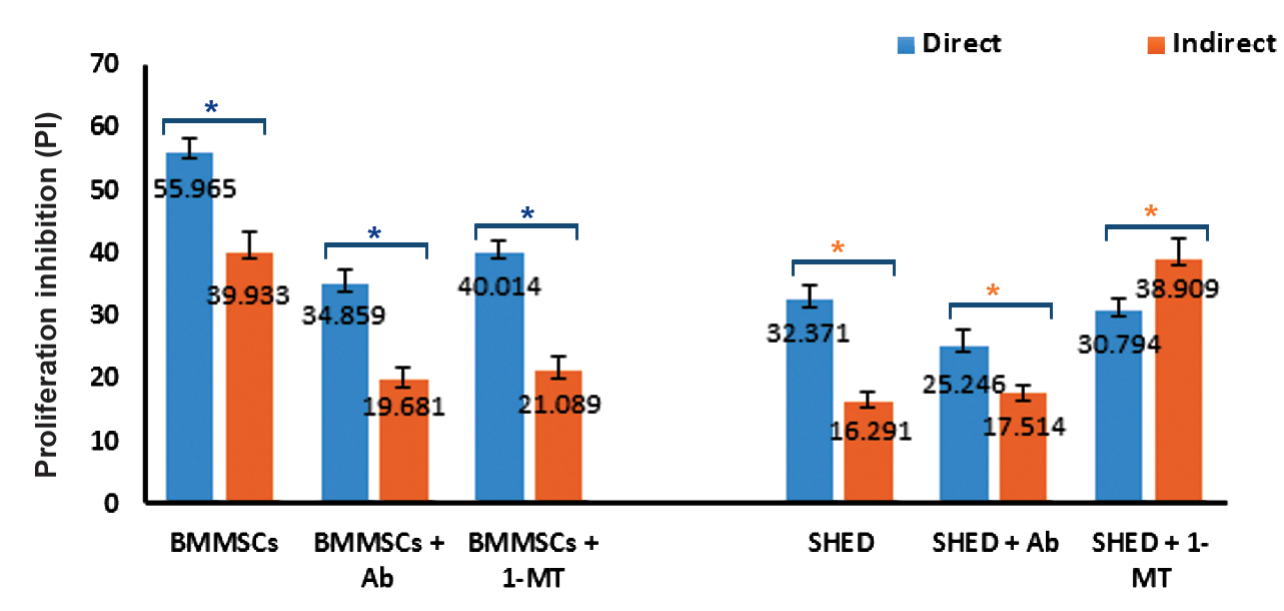
The effect of physical contact on immunosuppression of mesenchymal stem/stromal cells (MSCs). Inhibition of bone marrow mesenchymal stem cells (BMMSCs) and stem cells derived from human exfoliated deciduous teeth (SHED) in physical contact with (direct) and separate from T cells (indirect) in the simple co-cultures, Ab-co-cultures [4 μg/ml neutralizing anti-IFN-gamma (anti-IFN-γ) antibody] and MT-co-cultures [1 mM 1-methyl tryptophan (1-MT)]. Mean ± SEM of the results of two experiments performed in triplicate, were shown. Asterisks indicate significant differences (one-way ANOVA, P<0.05) between indicated groups. *; BMMSCs and *; SHED.

It has been reported that physical contact between MSCs and lymphocytes can play a role in their immunoregulation. Thus, we have co-cultures in normal cell culture plates that MSCs were in direct contact with stimulated T cells and co-cultures in Transwell plates where MSCs were isolated by a permeable membrane from T lymphocytes. We observed that BMMSCs in direct contact with T lymphocytes had stronger inhibitory effects on T cell proliferation, IDO activity, and cytokine production. However, for IL-2 and IDO activity, the results were not statistically significant for all cases (Fig .4, Table 2). Physical contact showed approximately the same effect on SHED co-cultures, with the exception of the MT-co-cultures where SHED showed more suppression when separated from T cells. There were no significant changes in IDO activity in the three cases (Fig .4, Table 2).

**Table 2:** The concentrations of IL-2 and IFN-γ according to ELISA, and kynurenine (KYN) by a colorimetric method in the three set of co-cultures in consideration of physical contact of T cells to mesenchymal stem/stromal cells (MSCs). The results are the mean ± SEM of two experiments (each performed in triplicate).


Co-culture	Contact	BMMSCs			SHED		
		IL-2	IFN-γ	IDO activity	IL-2	IFN-γ	IDO activity

Simple	Direct	33.14 ± 0.54	19.02 ± 2.48	15.75 ± 0.34	59.14 ± 4.43	33.87 ± 2.51	14.79 ± 0.23
	Indirect	40.64 ± 0.63	33.83 ± 1.54	14.65 ± 0.43	85.77 ± 3.76	60.19 ± 3.31	16.02 ± 0.32
	Sig.	0.227	0.00	0.00	0.00	0.00	0.071
Neutralizing anti-IFN-γ antibody	Direct	36.55 ± 0.24	17.81 ± 0.52	14.63 ± 0.24	93.35 ± 3.57	34.73 ± 4.04	13.54 ± 0.24
	Indirect	50.75 ± 2.74	14.50 ± 1.18	13.60 ± 0.46	63.25 ± 2.60	42.86 ± 1.12	14.11 ± 0.28
	Sig.	0.00	0.00	0.00	0.00	0.00	0.142
1-MT	Direct	46.51 ± 2.74	68.05 ± 4.50	12.95 ± 0.34	53.06 ± 1.62	36.30 ± 0.84	11.73 ± 0.68
	Indirect	51.32 ± 2.59	54.01 ± 6.26	13.62 ± 0.34	31.10 ± 1.12	27.88 ± 1.16	12.83 ± 0.33
	Sig.	0.449	0.055	0.164	0.00	0.00	0.274


BMMSCs; Bone marrow mesenchymal stem cells, SHED; Stem cells derived from human exfoliated deciduous teeth, IFN-γ; Interferon gamma, IDO; Indoleamine 2,3-dioxygenase, KYN; Kynurenine, 1-MT; 1-methyl tryptophan, and Sig.; Significance, one-way ANOVA, P<0.05.

## Discussion

Experimental data indicate that IDO activity induced preferentially by IFN-γ mainly contributes to the immunosuppressive effect of MSCs (25, 26). However IFN-γ is also considered to be one of the important factors that affect the immunoregulatory properties of MSCs (12, 18).

SHED are recently discovered compared to other MSCs (19, 22). Despite numerous studies concerning the role of the IFN-γ-IDO axis in immunosuppression of various MSCs, we have not found any publications that discuss the role of this axis in the immunoregulation of SHED. A better understanding of SHED immunomodulation will offer an insight into their use for clinical applications. Therefore, in this study, we have explored the role of IDO and IFN-γ in immunoregulatory effects of SHED and compared them to BMMSCs as conventional MSCs.

IDO catalyzes tryptophan into KYN, which can either enter the blood or additionally metabolize to further KYN metabolites which, in turn, exert immunoregulatory properties (16). Measurement of KYN concentration by an indirect colorimetric method is frequently used as an easy, acceptable method that estimates IDO activity (27-29).

The results showed notable activity of IDO in both MSC cultures (background controls), compared to the positive and negative controls that contained no MSCs. This observation indicated that either SHED or BMMSCs might produce functional IDO under normal conditions. The IDO activity between the two MSC cultures (two background controls) was similar.

Numerous studies have inconsistently reported that MSCs did not express IDO under basal culture conditions. Ryan et al. (17) and DelaRosa et al. (30) detected neither the expression of IDO protein nor IDO activity in the supernatant of human BMMSCs and human adipose-derived MSCs (hASCs) before IFN-γ treatment. However, other investigations implied that MSCs continuously expressed this enzyme in the absence of stimuli. According to results reported by Yoo et al. (31) the RNA of IDO was detectable in untreated hASCs. Additionally, Chang et al. (32) reported the IDO protein in two untreated human MSC populations, BMMSCs and placenta-derived multipotent cells (PDMCs). Similarly, Djouad et al. (33) detected clearly IDO activity in the supernatants of primary human MSCs.

This discrepancy could be explained somewhat by the heterogeneous nature of MSCs (34). Distinct BMMSC subsets that differ in their immunophenotype, morphology (35), and immunosuppressive action (36) have been reported. It has been well established that the clinical features of the subjects from whom the cells were isolated affect the characteristics of hMSCs (37). Evidences showed that either the culture or manipulation could impact the biological properties of MSCs (38, 39). Thus it is not unexpected that under the current study culture conditions, the two MSC groups (SHED and BMMSCs) produced functional IDO that caused detectable increased KYN concentration in their supernatants.

There was significantly increased IDO activity in co-cultures of both SHED and BMMSCs. Additionally in simple co-cultures, along with the increased MSC numbers that resulted in increased suppression, we observed increased IDO activity. As a conclusion from these data, IDO might contribute to the immunosuppression of both MSC types used in this research.

Next, we sought to confirm IDO involvement in immunosuppression of MSCs by the addition of 1-MT, an IDO inhibitor to the co-cultures. In the MT-co-cultures of BMMSCs, 1-MT significantly restored the lymphocyte activation which agreed with our results from the simple co-cultures. Increased immunosuppression was accompanied with augmented IDO activity in the simple cocultures. The inhibition of IDO activity was followed by decreased immunosuppression in the MT-co-cultures. Thus, our findings represented a key role of IDO in BMMSCs-induced immunosuppression which has been demonstrated in previous studies (40-42).

Unpredictably, in the MT-co-cultures with SHED, blocked IDO activity by 1-MT did not reduce immunoinhibition; rather, there was a remarkable increase.

This unexpected outcome contrasted numerous reports on various MSCs (25, 31, 32). However we have been unable to find any study on SHED that evaluated IDO. These results, however, need additional investigations. The following points are noteworthy.

IDO is known as an immunoregulatory enzyme (43) that participates in immunosuppressive events such as the escape of tumor cells from host immune surveillance (44) and allogeneic fetal tolerance (45).

However some reports (46) question the immunosuppressive nature of IDO. For example, in patients with systemic lupus erythematosus (SLE) as the disease progresses there is increased IDO activity in the blood (47). SLE is an autoimmune disease alleviated by immunosuppressants, hence, there is no explanation as to why IDO activity parallels disease exacerbation. Similar results have been reported in rheumatoid arthritis (RA) patients (48). Scott et al. (49) observed that when IDO activity was inhibited by subcutaneous administration of 1-MT in a mice model of RA, the disease was alleviated. 1-MT is expected to aggravate the disease by blocking the immunosuppressant IDO enzyme. IDO has been shown to aggravate inflammation in airways in animal models of allergic inflammation (50). These studies imply that IDO is not permanently immunosuppressive and it may have immune stimulatory effects under currently unknown conditions.

Recently, it has been revealed that beside the catalytic function of IDO as an enzyme, it also contains immunoreceptor tyrosine-based inhibitory motifs (ITIMs) which can bind to diverse molecular partners and affect intracellular signaling pathways (51, 52). Orabona et al. (53) have shown that under defined conditions IDO binds to SOCS3 (an intracellular signaling molecule) and the resultant IDO-SOCS3 complex is subsequently degraded.

In the SHED co-cultures, there might have been conditions under which IDO did not play an immunosuppressive role. However, we do not know the exact conditions responsible for this role. However, it is a weak possibility that 1-MT may play an unknown role in SHED. Some clinical researches suggest that the D isomer of 1-MT (D-1-MT) can play a role other than inhibition of IDO (54). This isomer (D-1-MT) does not participate in the inhibition of IDO activity, however, it enhances immunity in cancer patients (55, 56). Of note, in the current study, we have used a mixture of both the D and L 1-MT isomers.

According to the results of this study, we cannot completely explain the increase in immunoinhibitory action of SHED in the MT-co-cultures. Additional research is necessary to confirm and elucidate this result.

It has been established that the immunomodulatory capacity of MSCs is induced or at least augmented under inflammatory conditions (6-9). In this context, various methods have been used to examine the effect of IFN-γ as an inflammatory cytokine on the immunoregulatory action of MSCs. IFN-γ has a dual role. It is one of the first cytokines secreted from activated T cells and promotes their activation on one hand. However, on the other hand, IFN-γ induces MSC immunosuppression and enables them to more efficiently inhibit T lymphocyte activation (12). In this study, we have attempted to remove IFN-γ which was secreted by cells in the co-cultures (and is not exogenous) to levels that enabled T cell activation, yet limited immunosuppression of MSCs as low as possible.

The results showed that Abs caused a meaningful decrease in both the proliferation and cytokine production of stimulated T cells in the Ab-co-cultures of BMMSCs. Thus IFN-γ must have an enhancing effect on immunoregulation of BMMSCs. This finding was compatible with reported results from numerous researchers in terms of BMMSCs (57, 58) and other types of MSCs (12, 17). Although the results of IFN-γ SHED co-cultures showed a decrease in immunosuppression, we did not observe any significant decrease in T cell proliferation or cytokine production (IL-2). We found no study on SHED to compare our results. However, immunoregulatory actions of oral MSCs such as gingiva-derived MSCs (GMSCs) (59), periodontal ligament stem cells (PDLSCs), and dental pulp stem cells (DPSCs) (60) definitely increase in the presence of IFN-γ.

Our results did not completely agree with these studies. This might be due to inherent differences in various MSCs types and the individual characteristics of SHED.

Nevertheless, immunoinhibitory characteristics of SHED might be affected by IFN-γ. However because of its differences from BMMSCs, the conditions (for example the amount of IFN-γ) in which they are affected vary from BMMSCs. It has been identified that different tissue-derived MSCs respond differently to cytokines (61,63). This possibility was potentiated by our observation that with a SHED/T cell ratio of 1:10, the reduction in inhibition was more than expected and did not follow the trend shown in other ratios. It showed a decline compared to other ratios. Erkers et al. (64) showed that in contrast to BMMSCs, low levels of IFN-γ had no effect on the suppressive capacity of decidual stromal cells (DSCs). 

As a whole, we cannot conclude with certainty that IFN-γ did not affect immunoregulation of SHED. We expected that if other concentrations of IFN-γ or other methods such as pretreatment of SHED by IFN-γ were applied, SHED might be affected by IFN-γ. Next, we evaluated the IDO activity in Ab-co-cultures to check the IFN-γ-IDO axis in immunoregulation of MSCs. 

In the current study, for BMMSCs along with a significant reduction in IFN-γ by neutralizing Abs, we observed significant decrease in IDO activity in the co-cultures, which was followed by a significant diminish in immunoinhibition. A comparison of Ab-co-cultures to 1-MT-co-cultures showed that although the Ab had a more reductive effect, however as a whole, the two co-cultures did not significantly differ in terms of lymphocyte proliferation and cytokine secretion. Similar results were previously reported in which IFN-γ induced IDO play a major role or at least one of the important roles in immunosuppression of human BMMSCs (58,63). 

For SHED, we observed that the drop in IFN-γ in the Ab-co-cultures coincided with a decline in IDO activity. However, the reduction of IFN-γ in supernatants and the decrease in IDO activity did not accompany a significant decrease in immunosuppression. Therefore, it seemed that IFN-γ could increase IDO activity in SHED as well as in numerous other MSC populations. However, this enzyme does not mainly participate in immunosuppression of SHED. Rossi et al. (65) observed that human amniotic tissue derived MSCs (hAMTC) produced IDO. Although 1-MT could prominently reduce IDO activity, hAMTC induced immunosuppression did not change. Obviously this should be confirmed by similar experiments and further genetic studies. 

Finally, the evaluation of direct and indirect co-cultures (simple-co-cultures, Ab-co-cultures, MT-co-cultures) showed that in all cultures with physical contact between immune and stem cells, there was stronger immunosuppression. This was justified by the fact that some contact-dependent mechanisms also had a role in immunoregulation of MSCs (31,64). However, the results of the SHED MT-co-cultures were inverse, which should be further investigated. According to Nasef et al. (66), when MSCs have direct or indirect contact with immune cells, they might apply separate soluble immunoregulatory factors that affect these cells. Thus, in the MT-co-cultures, SHED probably produced factors with more immunomodulatory effects when separated from T lymphocytes compared to when they were in physical contact. MT-co-cultures of SHED showed different individual results compared to BMMSCs. 

## Conclusion

This study demonstrated that SHED as a subset of oral MSCs has immune properties that resemble other MSCs. SHED are similar to conventional MSCs or BMMSCs. They have anti-proliferative effects on stimulated T cells and reduce cytokine production from them. These properties of SHED may be affected by inflammatory conditions that occur in the presence of IFN-γ. However, we observed a non-significant decrease in immunosuppression of SHED after the use of neutralizing anti-IFN-γ antibodies, which differed from BMMSCs. SHED produced immunoregulatory factors such as IDO. Similar to BMMSCs, at least one of the inducers of IDO in SHED is IFN-γ. However unlike BMMSCs, this molecule does not mainly contribute to their immunosuppression and can have other cell-type specific roles. The IFN-γ-IDO axis seems to exist in SHED but it has no remarkable role in immunoregulatory effects. Finding the molecules involved in immunomodulatory effects of SHED and their differences with other MSCs requires additional research. 
